# Cutaneous Larva Migrans Presenting with Folliculitis

**DOI:** 10.4269/ajtmh.19-0949

**Published:** 2020-05

**Authors:** Mark Lander, Anna M. Checkley, Stephen L. Walker

**Affiliations:** 1 University College London Hospitals NHS Foundation Trust, London, United Kingdom;; 2 Hospital for Tropical Diseases, University College London Hospitals NHS Foundation Trust, London, United Kingdom;; 3 London School of Hygiene and Tropical Medicine, London, United Kingdom;

A 28-year-old woman returned from a scuba-diving holiday in Thailand with an intensely itchy rash on her anterior abdominal wall and buttocks. This started as a single pustule but developed into an extensive eruption of pruritic papules. Three days later, both buttocks were affected. She was otherwise well and had no fever, cough, dyspnea, or diarrhea. There was no response to oral flucloxacillin which was prescribed by a primary care physician.

**Figure 1.**
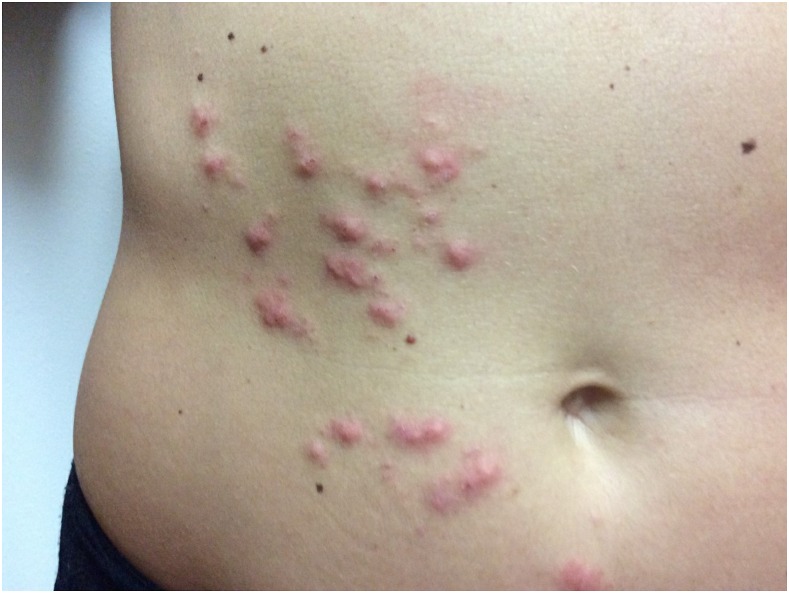
Follicular rash with occasional serpiginous tracts on the right anterior abdominal wall. This figure appears in color at www.ajtmh.org.

She presented to our emergency clinic, and on examination, there were follicular papules with occasional serpiginous tracts on the right anterior abdominal wall (Figure 1) and both buttocks. There was no dermographism, burrows, or lymphadenopathy. She had a peripheral eosinophilia of 3.77 × 10^9^/L (0.0–0.4 × 10^9^/L). HIV serology was negative.

**Figure 2.**
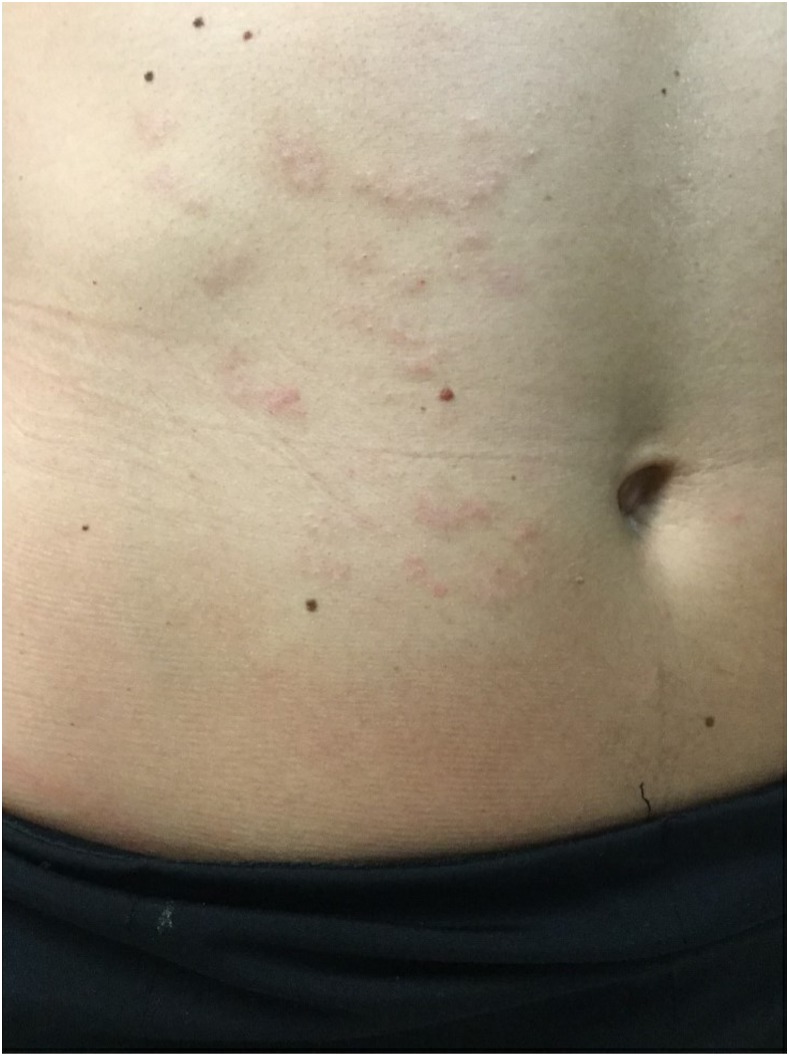
Resolution of the right anterior abdominal wall follicular rash. This figure appears in color at www.ajtmh.org.

A diagnosis of follicular cutaneous larva migrans (CLM) was performed, and she was treated with a single dose of oral ivermectin (200 µg/kg). The pruritus settled within 4 days and the palpable eruption 10 days later. On review 1 month later, the rash had faded, leaving macular post-inflammatory erythema (Figure 2), and her eosinophilia had resolved.

Cutaneous larva migrans is a common skin infestation in travelers returning from tropical destinations,^1^ although it can be acquired in cooler climates and even in the United Kingdom.^2^ The eruption is caused by larvae of various hookworm nematodes, which usually live within the intestines of cats and dogs, penetrating and migrating into the skin. *Ancylostoma braziliense* is the commonest pathogenic hookworm in CLM. Cutaneous larva migrans usually presents as a characteristic pruritic, migratory, serpiginous track or “creeping eruption.”

Follicular CLM is rare and may not be readily recognized.^3^ Papules may have a serpiginous distribution, and subtle tracks between papules may be identifiable. Follicular CLM often occurs on the buttocks or thighs.^3^ The pathogenesis is thought to be due to the immune response to the invading larva within the follicular canal.^4^ Follicular CLM responds well to ivermectin, whereas some authors suggest “classical” CLM has less response to ivermectin.^5^

It is important for clinicians who work in areas where CLM is common or see travelers returning from such areas to recognize this unusual clinical presentation.
